# Alignment of Molecular Classification Between Diagnosis and Recurrence in Endometrial Cancer: Lessons from a Single-Institution Experience to Inform Future Pathways

**DOI:** 10.3390/cancers18020247

**Published:** 2026-01-13

**Authors:** Stefano Restaino, Giulia Pellecchia, Martina Arcieri, Laura Mariuzzi, Maria Orsaria, Angelica Tulisso, Daniela Cesselli, Michela Bulfoni, Alice Poli, Federico Paparcura, Giorgio Bogani, Andrea Mariani, Gianfranco Zannoni, Giovanni Scambia, Giuseppe Vizzielli

**Affiliations:** 1 Clinic of Obstetrics and Gynecology, “Santa Maria della Misericordia” University Hospital, Azienda Sanitaria Universitaria Friuli Centrale, 33100 Udine, Italy; stefano.restaino@asufc.sanita.fvg.it (S.R.); giulia.pellecchia95@gmail.com (G.P.); giuseppe.vizzielli@uniud.it (G.V.); 2 PhD School in Biomedical Sciences, Gender Medicine, Child and Women Health, University of Sassari, 07100 Sassari, Italy; 3 Department of Medicine, University of Udine, 33100 Udine, Italy; daniela.cesselli@uniud.it (D.C.); michela.bulfoni@uniud.it (M.B.); 4 Institute of Pathology, “Santa Maria della Misericordia” University Hospital, Azienda Sanitaria Universitaria Friuli Centrale, 33100 Udine, Italymaria.orsaria@asufc.sanita.fvg.it (M.O.);; 5 Gynaecological Oncology Unit, Fondazione IRCCS Istituto Nazionale dei Tumori di Milano, 20133 Milano, Italy; giorgio.bogani@istitutotumori.mi.it; 6 Department of Obstetrics and Gynecology, Division of Gynecologic Surgery, Mayo Clinic, Rochester, MN 55905, USA; mariani.andrea@mayo.edu; 7 Gynecopathology and Breast Pathology Unit, Department of Woman, Child and Public Health, Fondazione Policlinico Universitario Agostino Gemelli IRCCS, 00168 Rome, Italy; gianfranco.zannoni@policlinicogemelli.it; 8 Gynecologic Oncology Unit, Department of Woman, Child and Public Health, Fondazione Policlinico Universitario Agostino Gemelli IRCCS, 00168 Rome, Italy

**Keywords:** molecular biology, endometrial cancer, recurrence

## Abstract

**Introduction**: Endometrial carcinoma (EC) is the most prevalent gynecological cancer. It is characterized by a clinical, pathological, and prognostic trajectory that has become inextricably linked to the disease’s molecular profile. Therefore, it is imperative to examine its relevance across all facets associated with the disease. **Methods**: This is a single-center retrospective study to assess tumor molecular profile concordance between EC diagnosis and recurrence. All patients who underwent hysterectomy for EC between 2016 and 2020 were included. **Results**: In total, 221 cases of EC were collected. In total, 18 recurrences were found. In two cases, there was a molecular classification (MC) change: an MMR-deficient endometrioid EC shifted to a “multiple classifier” subtype. The second, an NSMP subtype, at second recurrence revealed a switched MC to an aberrant mutated p53 profile. This discordance rate was non-significant in our cohort. However, considering the lack of evidence, it opens new insights to be revealed. **Conclusions**: This is the first study focusing on the discordance rate of MCs in EC relapses compared to the initial diagnosis. Future large-scale retrospective and prospective multicenter studies are essential for exploring this aspect.

## Simple Summary

Endometrial cancer treatment and prognosis have greatly improved thanks to advances in understanding its molecular profile. However, it is still unclear whether these molecular characteristics remain stable over time, particularly when the disease returns after initial treatment. This study explores the concordance and potential evolution of molecular classification between primary diagnosis and recurrence in endometrial cancer, building upon emerging evidence that has begun to address this question. This study explores the concordance and potential evolution of molecular classification between primary diagnosis and recurrence in endometrial cancer, building upon emerging evidence that has begun to address this question. By examining this relationship, our research provides valuable preliminary data that could guide future studies on the biological behavior of recurrent disease. These insights may ultimately contribute to more precise and personalized treatment strategies for patients with endometrial cancer.

## 1. Introduction

The current scientific and clinical approach to endometrial carcinoma is deeply rooted in molecular biology profiling for prognostic prediction and the growing application of precision medicine [1]. The newly updated FIGO 2023 staging system exemplifies and constitutes this shift. While it remains the subject of ongoing scientific debate, it has revised tumor staging by integrating and standardizing molecular classification (MC) alongside the tumor’s histopathological features to determine the final stage [2,3]. According to ESGO/ESTRO/ESP 2021 [4], MC is recommended to be conducted using the Cancer Genome Atlas (TCGA) surrogate, following the diagnostic algorithm by Vermij et al. [5]. This algorithm involves testing different immunohistochemical (IHC) markers (p53, MLH1, MSH2, MSH-6, PMS-2) and performing somatic mutation analysis of POLE (exons 9, 11, 13, 14) [6]. Guidance for interpreting the pathogenicity of POLE variants is provided by Leon-Castillo et al. [7]

If molecular classification data is available, it should be integrated into the conventional pathologic diagnosis [7]. The report must include details about the methods employed for both IHC and POLE mutation analysis, along with relevant literature regarding the pathogenicity of each identified POLE mutation [8]. These data should be incorporated alongside clinic-pathological information. The standard treatment for EC at diagnosis involves total hysterectomy with bilateral salpingo-oophorectomy, sentinel lymph node biopsy, and/or pelvic lymphadenectomy [9]. Recurrences are noted in approximately 20% of endometrioid cases and in 50% of non-endometrioid cases [5]. The management of this condition could benefit from greater consistency and a more robust dependence on the initial surgical intervention [10,11]. 

Additionally, it is essential to enhance our understanding of the significance of molecular factors in prognostic assessments, and observing their behavior from the first diagnosis onward to recurrence is crucial. Moreover, treating relapses requires a more detailed understanding of different therapeutic strategies to evaluate their prognostic and curative effects based on site and type of recurrence [12]. A recent systematic review of the literature began by analyzing the diverse group of recurrent endometrial carcinoma (EC) and highlighted the importance of choosing the right treatment according to the lesions’ location and whether they are single or multiple lesions. The authors concluded that the molecular patterns would be investigated in future studies to facilitate significant advancements in this clinical context. Currently, there are no precise indications, even from the latest NCCN 2024 recommendations, regarding the execution of MC at the time of relapse. However, recently published literature suggests its substantial clinical prognostic value [13].

This retrospective study seeks to evaluate a subset of patients with endometrial recurrence to establish a rationale for its use in this setting. Specifically, it examines whether the MC is concordant at the time of initial diagnosis and at recurrence in patients for whom MC data is available at both stages.

## 2. Materials and Methods

We performed a retrospective study of 221 cases of EC submitted to standard surgery at the Azienda Sanitaria Universitaria Friuli Centrale (ASUFC) from January 2016 to December 2020. All surgical procedures were performed at a single tertiary referral center, although surgeries were not conducted by a single surgeon. Standard surgical protocol for endometrial cancer was applied consistently throughout the study period, in line with contemporary national and international guidelines. Therefore, standard surgery consisted of total hysterectomy with bilateral salpingo-oophorectomy, with lymph node assessment performed according to risk stratification at diagnosis, but a sentinel lymph node was still not mandatory. Complete data regarding the type of surgery performed are available in Supplementary tables. Follow-up data were collected up to September 2023. The internal review board approved this study. All cases were collected from the registry of the Pathology Department at ASUFC and reviewed by a dedicated pathologist to obtain the following information: histotype according to the WHO, histopathological grading based on WHO and the 2023 FIGO staging system, myometrial invasion, presence of lymphovascular space invasion (LVSI), lymph node metastasis, FIGO/TNM staging, tumor classification, and molecular biology classification [14]. Preoperative staging was performed according to institutional protocols, with transvaginal ultrasound used as first-line imaging in early-stage disease and MRI reserved for selected cases when additional anatomical detail was required.

Through histological and clinical data, all cases of recurrence in the study sample were identified. For each of these, a biopsy and/or surgical specimen from secondary debulking surgery was available, on which molecular biology was performed to determine MC. Secondary debulking surgery was considered in cases of isolated or oligometastatic recurrence, good performance status, and resectable disease, whereas biopsy alone was performed in patients with multifocal, unresectable disease, poor surgical candidacy, or when histological confirmation was required to guide systemic or radiotherapeutic treatment.

Treatment selection at recurrence was guided by multidisciplinary discussion and by guideline recommendations available during the study period, without retrospective application of more recent updates.

To reduce the risk of technical variability or laboratory-related misclassification, all molecular analyses for both primary and recurrent tumors were retrospectively centralized and performed using the same immunohistochemical and molecular workflow. Standardized IHC protocols with internal controls were applied, and NGS analyses were conducted using UMI-based sequencing with predefined quality thresholds. Moreover, all paired samples were reviewed and classified by the same dedicated gynecologic pathologist, minimizing inter-observer variability. In particular, for the study’s aim, all identified cases of relapse were retrospectively re-analyzed by the same dedicated pathologist to perform immunohistochemical assays and complete molecular biology on both the samples of the first diagnosis and the recurrence because, at that time, the latter was not yet a standard procedure.

The sites of recurrence were categorized as follows:

- Locoregional recurrence: occurring in the vagina alone or in the pelvis, which may include simultaneous vaginal recurrence.
- Abdominal recurrence: this involves the greater pelvis, such as the pelvic sidewall, pelvic or paraaortic lymph nodes, and sigmoid colon, or the abdomen, affecting the liver surface, omentum, and abdominal wall.
- Extra-abdominal recurrence: distant recurrence happening outside the abdomen [14].

Molecular classification was performed by targeted next-generation sequencing (NGS) using a custom amplicon-based DNA panel with unique molecular identifiers (UMIs), specifically designed to detect hotspot mutations, including single-nucleotide variants (SNVs) and small insertions/deletions (indels) up to 20 bp. The assay enables the analysis of cancer-related genes. Sequencing was carried out on the Illumina MiSeq platform. Variant calling and alignment were performed using both CLC Genomics Workbench (v20.0.4, Qiagen, Hilden, Germany) and the GeneGlobe Data Analysis Center. For SNVs and small indels, variants were filtered with a threshold of 1% variant allele frequency (VAF), with an average coverage of approximately 500×.

For variant annotation and interpretation, the most updated releases of major reference databases were consulted, including dbSNP, ClinVar, COSMIC, OncoKB, MyCancerGenome, and the Clinical Knowledgebase (CKB, Jackson Laboratory, Bar Harbor, ME, USA v4.30). This comprehensive workflow ensured high-confidence detection, annotation, and classification of SNVs and small indels relevant to molecular classification. In accordance with the journal’s guidelines, we will provide our data for independent analysis by a selected team by the Editorial Team for the purposes of additional data analysis or for the reproducibility of this study in other centers if such is requested.

Statistical analysis: The most common test for comparing an observed percentage with a reference percentage is the test for a proportion. The formula of the z-test considers the two percentages, the obtained (rate of discordance) and a reference one (rate of concordance), and the sample size (number of recurrences). Using R software v4.4.2, we calculated the z-test and the *p*-value of the percentage of discordance found by two-tailed tests by applying the z-test to proportions. This latter test was used to define the statistical significance of the rate of discordance that emerged retrospectively [4].

## 3. Results

A retrospective collection of 221 consecutive patients diagnosed with endometrial cancer (EC) was conducted. A total of 18 recurrences were identified; of these, two patients received treatment for both the first and second recurrences.

Overall, 16/221 patients (7.2%) experienced at least one recurrence, accounting for a total of 18 recurrence events (18/221; 8.1%). Among these, 2/16 patients (12.5%) developed a second recurrence, while no patient experienced more than two recurrence events.

A total of 18 recurrence events were identified in 16 patients; two patients experienced two distinct recurrences each, while the remaining patients had a single recurrence. The clinical characteristics of the 16 patients with recurrence are summarized in Table 1.

The median age was 73 years, and the median body mass index (BMI) was 26 kg/m^2^. Regarding histology, 11/16 patients (68.7%) had low-grade endometrioid carcinoma (G1–G2), while 5/16 (31.3%) had high-grade histology. At diagnosis, the preoperative clinical stage distribution was: stage IA: 6/16 (37.5%), stage IB: 5/16 (31.2%), stage II: 1/16 (6.2%), stage III: 3/16 (18.7%).

All patients (16/16; 100%) underwent standard surgery; however, lymph node staging was absent in 50% of cases according to previous classifications for the initial disease stages. Further details, including preoperative radiological stage, a comparison with the final staging from FIGO2023, adjuvant therapies, and follow-up information, were gathered from clinical files and are summarized in Table 1 and Table 2. Please refer to Supplementary Tables S1 and S2 for additional case details at diagnosis. Adjuvant treatment was administered in 7/16 patients (43.7%), including: brachytherapy in 4/16 (25.0%), chemotherapy in 2/16 (12.5%), and combined radiotherapy and chemotherapy in 2/16 (12.5%).

More than 50% of the patients who experienced a relapse were initially diagnosed at an early stage (stages I–II according to the FIGO 2023 classification). Clinical data pertaining to the location of recurrence, as well as surgical and/or adjuvant therapies, were collected and summarized in Table 2.

Among the 18 recurrence events, the sites of recurrence were: locoregional: 10/18 (55.5%), abdominal: 6/18 (33.3%), extra-abdominal: 2/18 (11.2%).

Diagnosis of recurrence was established by biopsy alone in 11/18 cases (61.1%), while 7/18 (38.9%) patients underwent secondary debulking surgery. The second recurrence occurred in 4/18 recurrence events (22.2%), corresponding to 2/16 patients (12.5%).

Overall mortality among patients with recurrence was 7/18 (38.9%).

The surgical procedures varied, encompassing transverse oblique muscle resection, colon resection, and ureteral reimplantation, as delineated in Supplementary Table S3. Subsequent recurrence occurred in 2/16 patients (12.5%), accounting for 4/18 recurrence events (22.2%), for which molecular analysis was not available. The overall mortality rate among patients with recurrence was 7/18 (38.9%). The Supplementary Materials, specifically Tables S1–S3, provide additional details regarding the clinicopathological data.

Out of the 18 recurrence cases, molecular discordance between the primary tumor and recurrence was observed in 2/18 cases (11.1%), involving 2/16 patients (12.5%). One discordance occurred at the first recurrence, while the second was observed at the second recurrence. In one patient, molecular profiling revealed multiple variants, including POLE p.(Asp287As n) (11%), NRAS p.(Val14Ile) (14%), FH p.(Ala19Thr) (13%) and FH c.47G > A (7%), TP53 p.(Ala161Thr) (5%), CTNNB1 p.(Arg225His) (14%), and two KIT variants, p.(Asn512Asp) (30%) and p.(Gly663Arg) (33%). The low variant allele frequency of the TP53 alteration suggests that it is likely representative of a tumor subclone rather than a dominant molecular event. In the other patient, NGS analysis identified KRAS p.(Gly12Val) (21%), while TP53 alterations were limited to synonymous and intronic variants, without evidence of pathogenic mutations. Accordingly, TP53 status in this case should be considered wild-type from a functional standpoint. For this reason, p53 immunohistochemical findings cannot be directly equated with a copy number–high (CNH) molecular profile.

The first case involved a patient originally diagnosed with EEC G2, FIGO 2023 stage IA2 (negative LVSI and less than 50% myometrial infiltration). This patient underwent surgery, and no adjuvant therapy was necessary. Initially, the tumor’s molecular profile was categorized as “microsatellite instability” (MSI), indicating a deficiency in mismatch repair (MMRd). However, at the locoregional recurrence at the vaginal vault, a biopsy revealed a shift in molecular classification to a “multiple classifier” subtype, demonstrating both MSI and POLE exonuclease domain of uncertain significance (VUS) (p.Asp287Asn). In the second case, a first diagnosis identical to the previous one (EEC G2, IA2-FIGO2023, myometrial invasion <50% and negative LVSI), unknown lymph node status, no adjuvant therapy in the postoperative period, with a molecular profile at the presentation of CNL/non-specific molecular profile (NSMP) subtype. At the first locoregional recurrence on the vaginal vault, the diagnostic biopsy revealed stability of the molecular class, and the patient was treated with radiotherapy. After 4 years, a second locoregional recurrence occurred in the same vaginal site. The molecular classification in this case was switched to CNH (aberrant mutated p53) (Table 3). Statistical analysis showed a discordance rate of 11.1%, with a z-score of 1.51 and a *p*-value of 0.13, indicating no statistically significant deviation from concordance at a significance level of α = 0.05. In the first instance, no therapeutic intervention was conducted, and only follow-up evaluations were performed; conversely, in the second instance, therapeutic radiotherapy was administered upon the first recurrence episode.

## 4. Discussion

### 4.1. Summary of Main Results

Our retrospective study aimed to analyze the concordance rate regarding the MC between diagnosis and recurrence in EC. This study is the first to focus specifically on this issue to date. Given that MC has already demonstrated its prognostic significance in EC staging to the extent that it has been incorporated into the latest FIGO staging system, we are inclined to propose a new perspective regarding the identification of changes during the recurrence patterns. In our cohort, 18 recurrences (8%) were observed. Preoperative staging was performed with transvaginal ultrasound. Recent evidence indicates that expert transvaginal ultrasound and MRI show comparable diagnostic performance in the assessment of early-stage endometrial cancer, supporting the role of ultrasound as an effective first-line staging tool [15]. Moreover, advanced ultrasound techniques have demonstrated utility not only in local staging but also in the detection of lymph node involvement, further reinforcing their value in preoperative risk stratification [16].

In the POLE case, the identified mutation was POLE p.Asp287Asn (VAF 11%), a missense substitution located in the exonuclease domain of the gene. Although it is not among the canonical pathogenic hotspot mutations, its presence within the exonuclease domain suggests a potential functional impact on the proofreading activity of POLE, which may contribute to the hypermutated phenotype. This variant has not been reported in reference databases and currently lacks definitive functional characterization; it can therefore be considered a variant of uncertain significance (VUS). The interpretation of POLE exonuclease domain variants represents a well-recognized challenge in the molecular classification of endometrial cancer. While pathogenic POLE mutations are associated with an ultramutated phenotype and excellent prognosis, not all variants located within the exonuclease domain confer functional impairment. The POLE p.Asp287Asn variant identified in our cohort does not correspond to a canonical pathogenic hotspot and lacks functional validation; therefore, it should be considered a variant of VUS.

Integrative pathogenicity assessment tools, such as the scoring system proposed by León-Castillo et al., combine mutation type, tumor mutational burden, and mutational signatures to distinguish pathogenic from non-pathogenic POLE variants. According to these criteria, the available data for this case do not support definitive classification as a POLE-mutated tumor. Consequently, this case is best interpreted as a multiple-classifier tumor (MMRd with POLE VUS), highlighting the biological complexity of recurrent disease and the importance of cautious interpretation of molecular shifts over time [9,17].

Nevertheless, its detection in the context of a dMMR background and the observed clonal expansion at recurrence support the possibility that it may act as a driver rather than a mere passenger alteration, although additional studies, including ultra-deep sequencing and functional assays, would be required to confirm its pathogenic role [18].

### 4.2. Results in the Context of Published Literature

Reported recurrence rates in the existing literature range from 13% to 18%, despite the application of adequate recommended treatment, with rates of 20% for the endometrioid histotype and 50% for aggressive histotypes [9,19]. Apart from defining metastatic lesions potentially associated with the single MC profile [9], its value in the context of recurrences and how tumor’s genetic mutations could evolve over time has yet to be defined. Meanwhile, retrospective data on large case studies are needed to lay the foundations for further investigation.

MC also holds prognostic significance in recurrence and metastasis. In fact, McHenry et al. have recently evidenced that, when categorized by molecular subtype, disease-specific survival from the onset of high-stage presentations (stages III–IV) or from the point of recurrence in low-stage cases (stages I–II) among metastatic and/or recurrent endometrioid endometrial carcinoma (EEC) is strongly correlated with The Cancer Genome Atlas (TCGA) classification [5]. Although rare subclonal alterations may occur, this underscores the necessity of re-evaluating the TCGA classification in the context of recurrent or metastatic tumors [20]. In their study sample, a discordance of MC between primary and metastatic/recurrent tumors was observed in 4 out of 105 patients (3.8%); 2 of these cases were associated with PMS2/MSH6 IHC and the other 2 with p53 IHC [5].

A heterogeneous distribution of anatomical recurrence sites was observed across all molecular subtypes. No statistically significant differences in recurrence sites were found between the molecular subtypes, regardless of the stage. Furthermore, Moreno et al. identified mutations in recurrences that were absent in the primary tumor in 33% of cases and observed a greater number of mutational changes during the progression of Low Grade-EEC compared to High Grade-EC [21].

TGCA has established phenotypic/genotypic descriptors for each category: CNLs are usually EEC stable microsatellite tumours (G1, 2 and 3), low somatic changes in copy number, p53-wild-type (p53-wt) or few TP53 mutations with good-intermediate prognosis; CNHs conversely, are predominantly serous (94%) and mixed (62%) ECs, with a fraction of EEC (12%), and about 25% high-grade EEC, high copy number alterations [22]. Diametrically at the opposite extreme, there are EEC grade 3 tumours with POLE exonuclease domain mutations, with excellent prognosis even in high-grade tumours [20]. Another molecular category in its own right, both in terms of characteristics and prognostic value, is represented by multiple classifier tumors. This poses a challenge to the practical application of the molecular EC classification, as it is not immediately clear how these cases should be categorized or managed [23].

### 4.3. Strengths and Weaknesses

From our case history, despite the limitations imposed by the retrospective nature and the small sample size examined due to the monocentricity of the study, we identified two noteworthy instances of molecular discordance between the initial diagnosis and relapse. In one instance, the tumor acquired detrimental mutations that transitioned it from a CNL/NSMP class to a CNH/p53abn class, which is associated with a significantly worse prognosis. Conversely, in the other case, the change in molecular classification was attributed to the acquisition of the POLE mutation, which is recognized for its favorable prognostic and clinical attributes [24].

However, the POLE-EDM mutation detected at recurrence may indeed reflect clonal evolution from a pre-existing minor subclone in the primary lesion. Although our targeted sequencing panel has a detection sensitivity down to approximately 1% variant allele frequency (VAF), it is still possible that the variant was present at very low levels (<1%) in the primary tumor, below the technical limit of detection, and subsequently underwent clonal expansion during disease progression.

We recognize that tumor sampling can influence variant detection. Intratumoral heterogeneity may result in certain genomic alterations being confined to specific tumor regions.

Therefore, it cannot be excluded that the POLE mutation was already present but remained undetected due to the spatial sampling bias of the analyzed sections or the low frequency. A potential way to further investigate this hypothesis would be to reanalyze the primary tumor with ultra-deep sequencing approaches (sensitivity < 1% VAF), which could clarify whether the POLE variant was already present at very low allelic frequencies before recurrence. This would strengthen the interpretation that the molecular shift observed in the reported case reflects selection and enrichment of a pre-existing clone, rather than a truly novel mutational event acquired de novo.

Even the therapeutic implications of molecular classification at recurrence are further underscored by the advent of immune-checkpoint inhibitors. In particular, MMR-deficient endometrial cancers have demonstrated significant clinical benefit from immune-checkpoint blockade, which has now become a standard consideration in the recurrent setting. In the present series, immune-checkpoint inhibitors were not routinely administered, as most recurrences occurred before their widespread clinical availability. Nevertheless, these emerging therapies further support the importance of reassessing molecular profiles at recurrence to guide personalized treatment strategies [25].

Further research is necessary to interpret these findings adequately due to the requirement for additional analysis data [26].

Moreover, both cases presented low-grade histology at diagnosis: they were endometrioid EC G2 with negative LVSI in FIGO 2023 stage IA2; neither of them received adjuvant therapy at diagnosis.

Taking this into consideration, it can be postulated that the radiotherapy administered during the initial relapse may have led to the emergence of a therapy-resistant clone of mutated p53 tumor cells, which subsequently caused a second relapse at the same anatomical site. In this regard, while the five-year survival rate for low-grade, low-stage endometrial cancer (EC LGLS) stands at 95%, patients who experience a relapse encounter a poor prognosis coupled with limited treatment options. Despite the absence of aggressive histological or clinical characteristics, the underlying reasons for tumor recurrence remain ambiguous. In a recent pilot study, a subset of patients diagnosed with EC LGLS underwent genomic sequencing. The authors concluded that a genetic predisposition to recurrence may exist. Notably, three out of four recurrent cases exhibited copy number gains or amplifications, alongside significantly higher MSI sensor scores than non-recurrent cases. Furthermore, recurrent cases demonstrated a more significant tumor mutational burden in comparison to their non-recurrent counterparts [27]. Approaching this kind of surgery is also changing with minimally invasive surgery representing a feasible option for selected patients with recurrent endometrial cancer and who knows, perhaps MC will become a distinguishing factor [28].

### 4.4. Implications for Practice and Future Research

Until now, this study is the first to evaluate the degree of concordance between the molecular profiles of endometrial recurrences and the initial diagnoses. While the limited sample size and the results do not support further conclusions, they do raise questions warranting additional investigation: there are two contrasting scenarios that require further study through genomic sequencing [29]. What genomic and clinical features influence the acquisition of prognostically positive (case 1, POLE mutation) and negative (case 2, CNH/p53 abnormalities) molecular characteristic features needs to be further addressed, as other authors already supposed [30].

## 5. Conclusions

These results warrant validation and thorough investigation across a wide range of cases. This approach aims to customize treatment based on the molecular profile, even in the context of relapse. It is crucial to outline potential therapies that could serve as the framework, even within a limited set of cases, without excluding immunotherapy from this algorithm: a novel therapeutic landscape in endometrial cancer that has already become the standard of care.

## Figures and Tables

**Table 1:** Clinical Characteristics at Initial Diagnosis in Recurrent Cases.

Study Sample, N	16
Median age	73
Median BMI	26
Histology: N, (%)	
Low grade	11 (68.7)
High grade	5 (31.3)
Pre-operative clinical stage; N, (%)	
IA	6 (37.5)
IB	5 (31.2)
II	1 (6.2)
III	3 (18.7)
Surgery: N, (%)	16 (100)
Adjuvant treatment: N, (%)	
BRT	4 (25)
CHT	2 (12.5)
RT-CHT	2 (12.5)

Abbreviations: RT: radiotherapy; CHT: chemotherapy with carboplatin/paclitaxel, BRT: brachytherapy; Low grade: endometrioid G1-G2; High grade: endometrioid G3, aggressive histotypes.

**Table 2:** Details of Recurrent Lesions.

Sample of Recurrences: N (%)	18 (8)
Site of recurrence: N (%)	
Locoregional	10 (55.5)
Abdominal	6 (33.3)
Extra-abdominal	2 (11.2)
Surgery, N (%)	
Debulking	7 (38.9)
Only biopsy	11 (61.1)
Adjuvant treatment, N (%)	
EBRT	1 (14.2)
CHT	3 (42.8)
Other treatments (no previous surgery)	
EBRT	5 (45.4)
BRT	1 (9.0)
CHT	4 (36.3)
RT/CHT	1 (9.0)
Second recurrence, N (%)	4 (22.3)
Death, N (%)	7 (38.9)

Abbreviations: CHT was all carboplatin-paclitaxel except for one case of doxorubicin administration. EBRT: external beam radiotherapy; CHT: chemotherapy; BRT: brachytherapy.

**Table 3:** Molecular biology’s profile of endometrial cancer patients at diagnosis compared to recurrence.

Patient	Molecular Biology at Diagnosis	Molecular Biology at Recurrence
1	MSI	MSI
2	CNL	CNL
3	CNH	CNH
4	CNL	CNL
5	CNL	CNL
6	CNL	CNL
7	MSI	MSI
8	MSI	MSI
9	MSI	MSI
**10**	**MSI**	**MSI/POLE mut**
11	MSI	MSI
12 (a)	CNL	CNL
**12 (b)**	**CNL**	**CNH**
13	MSI	MSI
14	MSI	MSI
15 (a)	MSI	MSI
15 (b)	MSI	MSI
16	MSI: microsatellite instability MLH1: hypermetilated	MSI MLH1: hypermetilated

Abbreviations: CNL: copy number low; CNH: copy number high; MSI: microsatellite instability; MLH1: MutL protein homolog 1; POLE: polymerase epsilon (POLE) exonuclease domain. (b) Lowercase letters next to 12 and 15 cases refer to the second recurrence that happened in the same patient (respectively, patient 12 and patient 15).

## Data Availability

The data presented in this study are available on request from the corresponding author.

## Supplementary Materials

The following supporting information can be downloaded at: https://www.mdpi.com/article/10.3390/cancers18020247/s1, Table S1: Characteristics of patients at diagnosis; Table S2: Adjuvant therapy at diagnosis; Table S3: Characteristics of patients with recurrent EC.
