# Supplementary material for: Alignment of Molecular Classification Between Diagnosis and Recurrence in Endometrial Cancer: Lessons from a Single-Institution Experience to Inform Future Pathways
Source: Cancers (Basel). 2026 Jan 13;18(2):247. doi: 10.3390/cancers18020247 (PMC12839114; doi:10.3390/cancers18020247)
Supplement: Supplementary file 1 [file cancers-18-00247-s001.zip › cancers-4031552-supplementary.pdf]

Supplementary materials.

Table S1. Characteristics of patients at diagnosis.

Case	Age	BMI	Year of diagnosis	Histology	Surgery	Clinical stage	FIGO 2023 staging	Grading	Miometrial invasion	LVSI	Lymph nodal status
**1**	71	-	2015	Endometrioid	LPS	IA	IA2	G2	<50%	Pos	Nx
**2**	85	26	2016	Endometrioid	LPT	IA	IIC	G3	>50%	Pos, substantial	Nx
**3**	68	-	2016	Serous with focal clear cell	LPS	IA	IVB	G3	>50%	Neg	N0
**4 a-b**	67	30	2016	Endometrioid	LPT	IA	IA1	G1	<50%	Neg	Nx
**5**	54	24	2017	Endometrioid	LPS	IA	IIB	G1	<50%	Pos, substantial	Nx
**6**	78	24	2017	Endometrioid	LPT	IIIA	IIIA1	G3	>50%	Pos, substantial	Nx
**7**	74	24	2018	Endometrioid	LPT	IB	IA2	G1	<50%	Neg	Nx
**8**	65	-	2018	Endometrioid	LPS	IA	IA2	G2	<50%	Neg	N0
**9**	79	23	2018	Endometrioid	LPT	IIIC	IIIB	G2	>50%	Pos, substantial	Nx
**10**	74	25	2018	Endometrioid	LPS	IB	IA2	G2	<50%	Neg	N0
**11**	69	27	2018	Endometrioid	LPT	IB	IA1	G3	Absent	Neg	N0
**12a-b**	83	22	2019	Endometrioid	LPT	IA	IA2	G2	<50%	Neg	Nx
**13**	86	29	2019	Endometrioid	LPS	IB	IB	G2	>50%	Neg	N0
**14**	72	33	2019	Endometrioid	LPT	II	IIA	G3	>50%	Neg	N0
**15a-d**	70	27	2019	Endometrioid	LPT	III	IVA	G2	>50%	Pos	N0

**16**	82	34	2019	Endometrioid	LPT	IB	IIA	G1	<50%	Neg	N0

Legenda: LPS: laparoscopy, LPT: laparotomy, Nx: unknown nodal status; pos: positive; neg: negative; G1/2/3: grade;.

Table S2. Adjuvant therapy at diagnosis.

Case	Therapy (CHT, RT, HT)	If CHT, type and n° of cycles	If RT, n° of cycles	BRT	Dose (Gy)
**1**	BRT	-	-	-	30
**2**	None	-	-	proposed but rejected	-
**3**	CHT. RT was refused	Carboplatin, paclitaxel	-	-	-
**4**	none				
**5**	BRT	-	-	-	30
**6**	None, for PS ECOG.				
**7**	None				
**8**	None				
**9**	CHT+ HT (anastrozole)	-	-	-	-
**10**	None				
**11**	BRT	-	-	-	20
**12a-b**	none				
**13**	BRT	-	-	-	20
**14**	none	-	-	-	-
**15a-d**	CHT, RT	Carboplatin and paclitaxel, 4+2	25	-	45
**16**	CHT, RT	-	25	-	45+52.5

Legenda: CHT: chemotherapy; RT: radiotherapy; PS ECOG: performance status; HT: hormonal therapy: BRT: brachytherapy.

Table S3. Characteristics of patients with recurrent EC.

Case	Year of recurrence	Site of recurrence	Type of recurrence	Surgery at recurrence	Adjuvant treatment	CHT, cycles and type	RT, n° cycles, dose	BRT	Last Follow up	Second recurrence	Death from any cause
**12**	2021	Right inguinal lymph mass	Distant	Only biopsy	-	Carboplatin, paclitaxel, 3	-	-	09/2021	No	yes
**4**	2016	Vagina	Locoregional	Only biopsy	-	-	28, 56Gy	-	12/2020	Yes, CHT treated	Yes, 2021

**13**	2017	Posterior vaginal wall	Locoregional	Only biopsy	-	Doxorubicin, 2	-	-	01/2018	No	yes
**15**	2018	Bladder periureteral neoformation with right ureteral portion	Abdominal	removal of pelvic recurrence and right ureteral reimplantation	CHT	Carboplatin, paclitaxel, 6.	-	-	09/2024	No	no
**2**	2022	Para-aortic and pelvic lymph nodes	Abdominal	LPS: intercavoaortic, paracaval and right pelvic lymphadenectomy	CHT	Carboplatin and paclitaxel, 6 cycles	-	-	07/2024	No	no
**16**	2018	Vaginal cuff	Locoregional	Only biopsy	-	Carboplatin, single agent (1 cycle, then stopped for PD and intolerance)			10/2018	No	Yes
1	2020	Meso-rectal mass infiltrating rectus muscle tissue (recurrence on the right mesorectal plane about 5 cm from the perineal plane)	Abdominal	Supraumbilical-pubic LPT: resection of the rectum and packing of ostomy. RT=0	-	-	28, dose 50.4 Gy	-	04/2024	suspected	no
**3**	2021	Vagina	Locoregional	Xipho-pubic LPT: vaginal dome biopsy.	RT	-	33, 66Gy	-	07/2022	No	no

**5**	2018	Vaginal cuff	Locoregional	Only biopsy	-	-	Palliative RT, 25, 20Gy	-	05/2019	No	Yes 2019
**6**	2020	Vaginal dome + appearance of suspected pulmonary nodules not biopsied	Locoregional + suspected distant	Only biopsy	-	Carboplatin paclitaxel, 3 cycles	-	-	04/2021	No	Yes, 2022
**7**	2019	Biopsied bilateral breast metastasis + suspected non-biopsied axillary and left obturator lymph nodes	Distant	Only biopsy	-	Carboplatin, paclitaxel, 6; avelumab	-	-	09/2024	No	no
**8a**	2019	Vaginal cuff	Locoregional	Only biopsy	-	-	25, 45+52.5 Gy	-	06/2024	01/2023	no
**8b**	2023	Vaginal cuff	Locoregional	Only biopsy	-	-	5, 20Gy	-	06/2024	-	no
**9**	2021	Oblique transverse muscle	Abdominal	Diagnostic LPS and subsequent LPT with excision of subfascial neoformation at the left external transverse oblique muscle	-	-	-	-	05/2022	2022: lung metastases	no
**10**	2019	Anterior vaginal wall	Locoregional	Only biopsy	-	Carboplatin	-	-	09/2019	PD	yes

						paclitaxel, 4;					
**11a**	02/2021	Lateral pelvic (paravescical peritoneal neoformation, ascending colon)	Abdominal	LPT with viscerolysis, removal of right paravescical peritoneal neoformation, tangential resection of ascending colon, removal of right ovarian pedicle.	-	-	-	-	09/2024	07/2021 vaginal recurrence	no
**11b**	07/2021	Vaginal cuff	Locoregional	Only biopsy	-	Carboplatin, paclitaxel, 9	SBRT 5; 30Gy	-	09/2024	-	no
**14**	2021	Abdominal wall	Abdominal	LPT with removal of suprafascial neoformation of abdominal wall	CHT	Carboplatin, paclitaxel, 2	-	-	07/2024	No	No

Legenda: CHT: chemotherapy; LPS: laparoscopy; PD: progression disease; LPT: laparotomy.
